# SPEAK-SAFE: secure processing of electronic audio for knowledge in suicide assessment from therapeutic exchanges

**DOI:** 10.3389/fdgth.2026.1616955

**Published:** 2026-02-24

**Authors:** Christopher Landau, Patricia Getty, Caroline Gruler, Rebekka Stadje, Sofia Arampatzi, Aishik Mandal, Anmol Goel, Iryna Gurevych, Andreas Reif, Oliver Grimm

**Affiliations:** 1 Department of Psychiatry, Psychosomatic Medicine and Psychotherapy, University Hospital Frankfurt, Frankfurt am Main, Germany; 2 Ubiquitous Knowledge Processing Lab (UKP Lab), Department of Computer Science and Hessian Center for AI (hessian.AI), Technical University of Darmstadt, Darmstadt, Germany; 3 Fraunhofer Institute for Translational Medicine and Pharmacology ITMP, Frankfurt am Main, Germany

**Keywords:** artificial intelligence, German speech dataset, natural language processing, suicide risk assessment, therapeutic dialogue analysis

## Abstract

**Background:**

For therapists, the spoken word of their patients is among the most important foundations for clinical assessment. At the same time, it is hardly possible to monitor patients continuously and closely in sufficient numbers, for example, to ongoingly assess the risk of suicide in therapeutical conversations. Natural Language Processing (NLP) involves the use of Artificial Intelligence (AI) to analyze human language. Combining it with AI speech processing methods, we obtain multimodal methods which can automatically process large volumes of speech and language data to extract diagnostic information and therefore support individualized treatment plans. Thus, in NLP/multimodal methods, we see the opportunity to significantly improve patient care.

**Methods:**

The SPEAK-SAFE project, implemented by clinicians and clinical researchers from the University hospital in Frankfurt in collaboration with the AI experts from the TU Darmstadt, aims to create the first German psychiatric corpus for evaluating and developing multimodal and NLP models to optimize diagnostic processes in psychiatric, psychosomatic, and psychotherapeutic care. Therefore, we will collect therapist-patient dialogues during therapy sessions. This sensitive data necessitates robust privacy. To meet this requirement, all collected data is pseudonymized, to ensure that no personal data is part of the evaluation and training of the AI models.

**Discussion:**

During the implementation of our research project, we were faced with challenges regarding the security of patient privacy and the technical implementation of therapy recordings toreassure sufficient data quality for the data analysis. Therefore, in addition to improve the suicidality prediction with multimodal methods we will develop an end-to-end-workflow for further AI-research in the clinical context.

**Clinical Trial Registration**: https://drks.de/search/de/trial/DRKS00027878, identifier DRKS00027878.

## Introduction

### Background and rationale

According to the Federal Statistical Office of Germany suicide led to more than 10,000 deaths in 2024 (1) and is the most common cause of death among young people between the age of 10–25 in 2023 (2). In most suicide cases, individuals suffered from mental health disorders with rates ranging from 87,3% (3) up to 96,8% (4). Preventing suicides or suicide attempts is the goal of every psychiatric patient treatment and requires an ongoing suicide risk assessment. Thereby, most people committing suicide consult clinicians in the days or weeks prior to death (5, 6). However, predictive markers are currently not sufficiently able to predict suicide risk. Clinicians rely on spoken self-reports to gain insights into the patient's psychological state. Speech comprises a variety of aspects which carry information about someone's psychological state, like e.g., the content, speaking rhythm, loudness, pitch, pace, or coherence (7, 8). The diversity of content-related linguistic features that have been shown to be relevant for diagnostic assessment (9) and for the identification of suicidal ideation (10) is so extensive that a comprehensive evaluation of these features during an ongoing clinical conversation is unrealistic. It is therefore plausible that, along other factors, this linguistic complexity contributes to the limited consistency observed in suicide risk assessments (11). Further, some of the aspects of speech are subtle and hard to assess reliably by a human listener during a conversation, like e.g., jitter, shimmer, or harmonics-to-noise ratio, but important for the understanding of psychiatric illness' like depression (12). Thus, to implement meaningful preventive measures, there is an urgent need for methods able to assess all important predictive markers related to suicide risk and mental disorders in general. Recent advances in Natural Language Processing (NLP), a field within artificial intelligence (AI), show promising results in mental disorder detection and an upward trend within this research area (13). Within NLP methods, linguistics and computer science enable large-scale analysis of language which are also applicable to the clinical psychological practice, where language plays a crucial role in patient care. NLP can be applied to various kinds of text data like social media posts (14–16), interviews (17, 18), and clinical notes (7, 19, 20). For the clinical psychiatric context, studies show promising results for the appliance of NLP methods. Regarding the field of suicide and suicide risk research, NLP models can successfully identify patients who have attempted suicide (20), predict suicidal ideation (17), and distinguish between suicidal and non-suicidal teenagers (21). Furthermore, suicidal language appears to be a stable classifier when compared to standard measurements. This stability is evident as suicidal language persists for up to 30 days after discharge from care, even when responses to other measurements change (18). Accordingly, there are multiple NLP-based methods which enable the exploration of previously unexamined linguistic features.

The aforementioned approaches have been somewhat overshadowed since the introduction of the Transformer architecture (22) and its fast development and huge impact, the evaluation of speech data has totally changed. Models have grown tremendously in a short period of time e.g., GPT3 is more than 100 times bigger than its predecessor GPT2 (23). Due to this rapid development, it is now possible to use even large amounts of speech data, such as clinical interviews, for evaluation with large language models (LLMs). Modern LLMs are not only ideal for general purposes, they also show capabilities when it comes to specific tasks in the domain of mental health (24). It could be demonstrated that LLMs like GPT-3.5 and GPT-4 are able to correctly identify patients with depression. However, they are known to show a considerable language bias and due to their nonspecialized training data, they are better at identifying control participants which could narrow their direct applicability for mental health applications (25). Models specialized on medical knowledge like Med-PaLM-2 can demonstrate human-like performance when predicting PHQ8 scores used for the assessment of the severity of depression (26). Besides spoken language many studies utilize written language like social media data and show promising results even with simple prompting strategies (27). Modern LLMs capabilities go way beyond this and offer numerous applications in addition to those mentioned before. Other studies indicate their usefulness in giving treatment suggestions (28) or creating simulated patients for psychotherapy training (29).

Apart from the content it is also important to take acoustic features accompanying the linguistic content in speech, also called prosodic and intonational features into account. These features are beyond the content related aspects of speech like phonemes and comprise patterns of pitch, duration, intensity, and rhythm, but are important in conveying meaning, emotion and nuance in speech (30). Recent studies focusing on acoustic features (31, 32) confirm that detection and severity assessment of mental health conditions like depression can be achieved with high accuracy based solely on acoustic information, demonstrating that vocal markers provide a reliable proxy for underlying affective states. In addition to research focusing on one modality, there are studies that consider both linguistic and acoustic features in a single study (33).

With multimodal models, large scale language and acoustics data can be processed simultaneously to get a better prediction of e.g., suicidality (34) and depression (35). Those multifaceted insights can greatly enrich existing knowledge about diseases like depression or schizophrenia and thus improve patient diagnosis and care. Moreover, a multimodal approach enables a much more realistic evaluation of speech data sets and is therefore useful in many counseling situations.

## Objectives

Considering the lack of linguistic diversity in datasets with a clear bias towards English (13, 36) and social media data (24), we see an urgent need for further non-English speech datasets (37). To this day there is no sufficiently large German speech corpus from severely ill psychiatric patients. There have been several valuable efforts to advance the analysis of language in the context of psychotherapy and mental health within German-speaking populations. However, existing studies have primarily focused on outpatient samples with milder symptom severity (38) or relied on relatively small datasets (39). While these contributions are undoubtedly significant, they nevertheless leave an important gap regarding large-scale datasets from more severely affected patients in inpatient settings. Existing corpora, such as the Research and Teaching Corpus of Spoken German (FOLK) (40), are not specifically designed to meet the needs and peculiarities of psychiatric and psychotherapeutic contexts, thus limiting their utility for NLP and multimodal methods in these fields. Even from a global perspective, language data sets are rather rare compared to the multitude of existing languages (41).

We therefore aim to generate a large enough trans-diagnostic collection of audio data of hospitalized patients and patients treated in our outpatient clinic. A study by Parola et al. (42) has shown several language specific differences regarding reduced semantic coherence in schizophrenia, which indicates a limited generalizability of speech patterns across different languages.

With our study, we aim to fill this gap by applying recent advances in NLP and multimodal methods. Our goal is to help identify non-explicit content language patterns in clinical patients, which can serve as prognostic markers specifically for the German language. Thus, our primary aim is to assess the suicide risk in patients with depression, bipolar disorder, and psychotic disorders, such as schizophrenia, schizoaffective disorder and acute polymorphic psychotic disorder (according to ICD-10), as well as the psychopathological development (Clinical Global Impression, CGI). As a secondary outcome we want to assess the overall psychopathological condition of the patients. This includes standardized and validated questionnaires comprising the clinician's impression of the patients general psychosocial functioning (Global Assessment of Functioning, GAF), suicidal symptoms (Depressive Symptom Index—Suicidality Subscale, DSI-SS) and suicidality (Suicidal Scale of the Mini-International) as well as electronic health records (EHR) of the patient's psychopharmacological medication. Generally, the standardized text of the patient's EHR will be accessible for the text-based analysis. These will be supplemented by the standardized assessment through a clinician, which will be used as the gold standard for comparison with the AI analysis.

## Trial design

The study is multicentric, data will be collected via the University hospital in Frankfurt and in the Private Clinic Dr. Amelung in Königstein and will be processed by data scientists from the TU Darmstadt. The raw data will be stored exclusively on protected local servers at the University hospital Frankfurt and only pseudonymized data will be transferred to Darmstadt.

## Methods: participants, interventions and outcomes

### Study setting

Data will be collected in the University hospital in Frankfurt a.M. Additionally, one clinician and project member will collect data at the private hospital Dr. Amelung in Königstein. Data access is regulated via a data security protocol.

### Eligibility criteria

We will include patients with ICD-10 diagnoses F20.x, F23, F25, F30-33, aged between 18 and 65 years, who are legally competent, without any issues in voice production, and have a German speaking proficiency of at least B2 (lower level in German would contain too little information content of linguistic utterances). Additionally, psychotherapists employed at the University Hospital Frankfurt will be included for the therapeutic conversations.

### Who will take informed consent?

Informed consent will be contained by members of the study team who are not involved in the patients care. The patients are addressed at least one day prior to study participation and informed about the study. One day later, they will be addressed again and sign the consent if interested in participation.

## Interventions

### Explanation for the choice of comparators

n/a: No comparator treatment was conducted. All participants equally engaged in receiving therapy sessions and completing questionnaires.

### Intervention description

The participation will consist of the recordings of the therapeutic clinical conversations that are carried out in the regular clinical practice. In addition, for our study a student assistant will place a recorder and microphones before the therapy session and pick it up afterwards. This student assistant will be absent during the therapy session and only install the recording hardware. Further, after the recorded therapy session the clinicians and participants are required to fill out questionnaires regarding the clinical state and suicide risk of the patient on the same day as the therapy session was recorded.

### Criteria for discontinuing or modifying allocated interventions

Study participation will be discontinued if the patients or their therapist request to discontinue participation or if the patient's clinical treatment is completed.

### Strategies to improve adherence to interventions

To maintain adherence as much as possible, we strive to integrate the participation process seamlessly into the clinical routine of both patients and therapists. The only additional effort required for participation is approximately 5 min to fill out questionnaires after a recorded therapy session.

### Provisions for post-trial care

n/a: we do not expect that patients suffer any harm from participation, since the recordings are conducted as part of their regular therapeutic care and participants additionally only fill out questionnaires.

### Outcomes

As part of an initial exploratory analysis, the assessment of psychopathology by treating physicians is to be compared with the evaluation by a computer model. Therefore, using intraclass correlation coefficients or t- tests, the hypothesis “There is no difference in the assessment of patients regarding the severity of illness and suicidality” will be tested.

### Participant timeline

In-patients and out-patients will regularly be screened for participation eligibility. If the eligibility criteria are met and in consultation with their treating clinicians, patients will be informed about their study and will have at least one day to consider participation and sign the informed consent. The same procedure applies for clinicians who participate in the study, while they only must consent once for the recording of therapy sessions with several patients who gave informed consent. From the time point on where patients agreed to participate, up to five of their regularly held therapy sessions will be recorded. After the therapy session, patients and therapists will fill out questionnaires about the psychopathology of the patient (approx. 5 min. for each). They are asked to fill out the questionnaire on the same day the therapy session took place. Table 1 summarizes the procedure and when the respective questionnaires are used.

**Table 1:** Content for the schedule of enrollment and participation.

TIMEPOINT	Enrollment	Informed Consent	Recording and Questionnaires				
	*−t2*	*−t1*	*t1*	*t2*	*t3*	*t4*	*t5*
	Ongoing	After confirmation by clinician	At least 1 week between each participation				
			Up to five therapy sessions can be recorded per participant				
			Questionnaires have to be filled out the same day as the recording				
*Study admission*							
Eligibility screen	X	X					
Consultation with the treating clinician	X						
Informed consent		X					
*Recording of therapy session*			X	X	X	X	X
*Patient reported outcomes (PRO)*							
*Beck’s Depression Inventory (BDI-II)*			X	X	X	X	X
*DSI-SS-Scale*			X	X	X	X	X
MINI C Suicidality Scale			X				
*Demographics, Diagnoses and Concomitant Medication*			X				
*Therapist Ratings*							
*Global Assessment of Functioning (GAF)*			X	X	X	X	X
*Clinical Global Impression (CGI)*			X	X	X	X	X
*Brief Psychiatric Rating Scale (BPRS)*			X	X	X	X	X

### Sample size

To determine the required sample size for comparing the accuracy of our models against the gold standard of a trained psychiatrist's evaluation, we conducted an *a priori* power analysis using G*Power software (43).

We based our effect size estimate on the accuracies reported in previous studies that used machine learning techniques to predict suicidality from clinical data (44). Pestian et al. (45) reported an accuracy of 85%, Bernert et al. (46) reported 90% among 87 studies in their meta-analysis, and Barak-Corren et al. (47) reported 94%. The average accuracy across these studies is 89.2%. Assuming a gold standard accuracy of 100%, we calculated an effect size (Cohen's g) of 0.11 using the formula: g = (P—Po)/√[Po(1—Po)], where *P* is the expected accuracy (0.892) and Po is the gold standard accuracy (1.0).

For our power analysis, we set the following parameters: a two-tailed test, an alpha level of 0.05, a power of 0.80, and a constant proportion of 0.5 (representing a balanced dataset). The results indicated that a total sample size of 162 is required to detect an effect size of 0.11 with 80% power at a significance level of 0.05. The critical values for the lower and upper bounds of the acceptance region are 68 and 94, respectively. The actual power achieved with this sample size is 0.8046757, and the actual alpha level is 0.0491726.

In summary, to compare the accuracy of our models against the gold standard of a trained psychiatrist's evaluation with 80% power and a 5% significance level, we require a total sample size of *n* = 162, assuming an effect size of 0.11 based on the accuracies reported in previous comparable studies. As we expect a drop-out rate of 15%–20% due to technical issues, quality issues etc., we expect to collect *n* = 200 interviews.

### Recruitment

The participants are recruited from both inpatient and outpatient individuals at the Department of Psychiatry, Psychosomatics, and Psychotherapy at the University Hospital Frankfurt. Additionally, a physician collects data at the Dr. Amelung Private Clinic in Königstein. For ongoing enrollment, patients will be screened regularly for participation criteria by the study teams and by their treating clinicians. Study information will be given by members of the study team who are not involved with the patient's treatment to prevent participation out of a sense of obligation and to maintain voluntary participation. There will be no assignment of interventions since participation does not include differing intervention types.

## Assignment of interventions: allocation

### Sequence generation

n/a: since participation comprises the recording of up to five regularly held therapy sessions and subsequent filling out of questionnaires which will be equally done by all study participants, sequence generation is not needed.

## Data collection and management

### Plans for assessment and collection of outcomes

The recording of the therapy sessions and the questionnaire data will be collected on the same day. An overview of all applied study instruments is depicted in Table 2. After giving the patient the study information and obtaining informed consent, audio recordings are conducted during routine medical activities. Up to five of their therapy sessions with a typical duration of 30–60 min are recorded. Audio recordings of therapeutic sessions are captured using high-quality recording equipment with carefully positioned microphones to minimize background noise and ensure clear speech capture. Recordings are made using a Tascam DR-40X audio recorder. Recordings are captured in stereo mode using the recorder's external left/right inputs. The audio is stored as uncompressed WAV files with a sampling rate of 48 kHz and a bit depth of 24 bit. The recorder input level is set to −12 dB, providing sufficient headroom to avoid clipping. Phantom power (48 V) is enabled to supply the external microphones. Audio signals are acquired using two external condenser microphones (the t.bone CC 75) with a frequency response of 20 Hz–20 kHz, corresponding to the standard human auditory range. Microphones are attached at the chest/collar area of the participants ensuring comparable distance to the mouth between the participants. Care is taken to ensure that the microphone grille did not come into contact with participants' clothing and is oriented toward the participant's head, in order to minimize clothing-rub noise and to enable more accurate recordings. These settings were chosen based on previous test runs using different setups of microphones and recording environments/parameters. We transcribed and diarized the recordings and manually evaluated the quality of these transcriptions with their corresponding audio recordings to find the best setup.

**Table 2:** Overview of collected data.

Data category		Purpose of use	
Demographic data		Control of the study*’*s inclusion criteria	
Clinical data	Clinical Global Impression (CGI)	Dimensional measure of development of psychopathology	Assessment of the patient*’*s condition or psychopathology
	Clinical Global Impression of Suicide Severity (CGI-SS)		
	Global Assessment of Functioning (GAF)	Assessment of the general psychosocial functioning level	
	MiNi C Suicidality Scale (MiNi)	Assessment of suicide risk and preceded suicidality	
	Suicidality Scale of the Depressive Symptom Index (DSI-SS)	Measuring instrument for recording suicidality	
	Beck*’*s Depression Inventory (BDI- II)	Severity detection	
	Brief Psychiatric Rating Scale (BRPS)	Severity detection	
	Additional information obtained from medical record	Text based analysis	
	Medical history		
	Concomitant Medication		
Recording of the therapy sessions		Analysis of speech features	

Additionally, data on general psychosocial functioning and the severity of the patient's psychopathological state are collected using questionnaires completed by both therapists and patients.

The Clinical Global Impression (CGI) is a short assessment of the clinician's view of the patient's global functioning and to track patient progress or treatment response over time and comprises three measures of which we use two: severity of illness, global improvement, and an efficacy index (which we leave out) (48). The instrument has been proven to be a readily understood and practical measurement tool which is well applicable in the clinical practice (49) and has been used in different mental disorders including affective and psychotic disorders (50, 51). Additionally, we use the CGI-SS which is a modified version of the CGI to evaluate severity of and change in suicidality according to the version of Meltzer et al. (52). We only changed the time period from “past 7 days” to “since the last assessment”.

The Global Assessment of Functioning (GAF) scale is an established instrument for measuring illness severity in the clinical context and introduced as a rating scale within the DSM-III-R (53). The instrument has shown to be a valid and reliable measure of psychiatric illness (54).

### Plans to promote participant retention and complete follow-up

To promote retention of the participating patients and their therapists, we are integrating the participation progress in everyday clinical care with minimal extra effort for the participation process. If therapists or patients nevertheless want to end their participation, there will be no consequences or disadvantages.

### Data management

During the data collection phase, the raw audio recordings, patient data, and questionnaires will be collected. In this phase, only clinicians and student assistants from the University hospital in Frankfurt as well as one doctoral student from the Privatklinik Dr. Amelung in Königstein are involved. The analysis of the data is conducted by the Ubiquitous Knowledge Processing (UKP) Lab at TU Darmstadt using natural language processing and multimodal methods and is mostly carried out in Darmstadt exclusively with pseudonymized data. The data accessed by the research group from TU Darmstadt will only have pseudonymized files and data without any personal information about the participants. The data analysis is complemented by a standardized assessment of the course by an independent board-certified psychiatrist or experienced assistant in psychiatry, serving as a “gold standard” for comparison with the AI analysis. Thus, the pseudonymized audio files are listened to and classified by a board-certified psychiatrist based on clinical features. The gold standard assessment covers three different areas. It consists of the BPRS as a broad psychopathology questionnaire, the five-stage severity item of the CGI-SS combined with an open question, asking the experts to briefly justify their rating within 2–3 sentences and the VTAS-R-Short Form, a five-item therapeutic alliance questionnaire (55).

**Figure 1:**
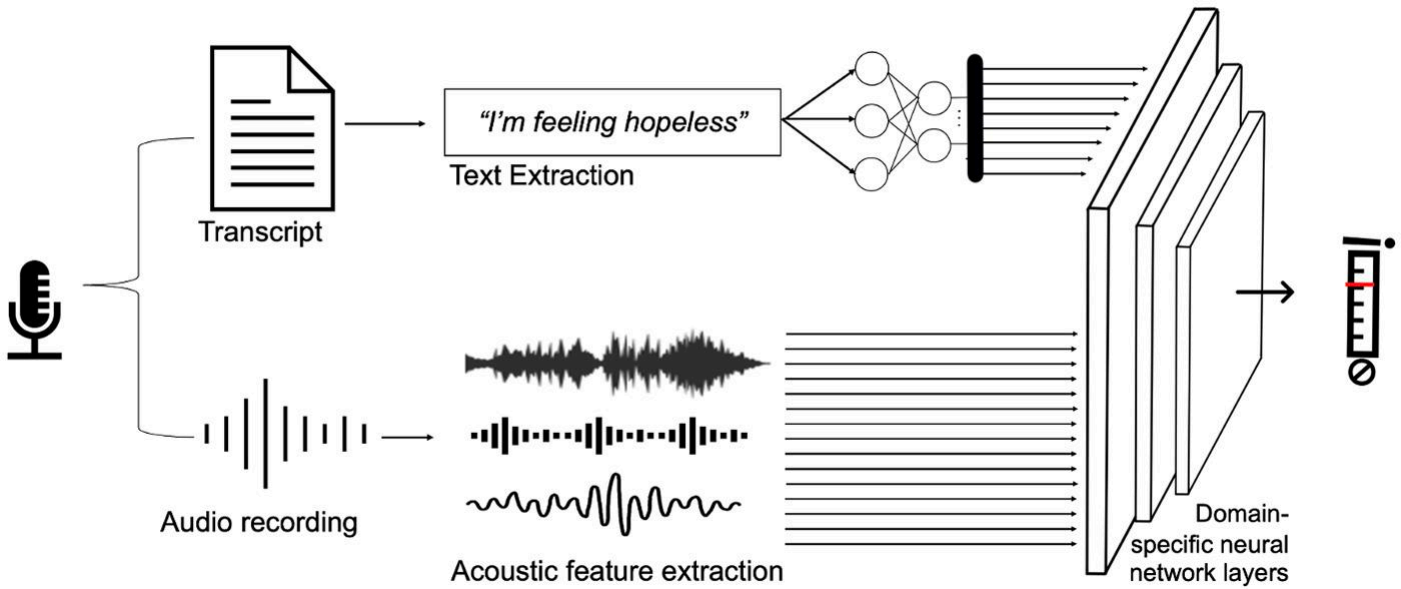
Schematic depiction of data collection and analysis workflow.

The pseudonymized audio recordings will then be analyzed for acoustic and prosodic features using the open-source toolkit, openSMILE (56) and then transcribed to text with the freely available software WhisperX (57). Both datasets will then be analyzed on its own and later on fed to a multimodal neural network to generate a clinically relevant outcome score (e.g., suicidality prognosis score). Figure 1 shows an overview of our workflow.

Both, audio track and therapy transcript have to be pseudonymized to ensure data privacy. The sessions are transcribed to text with the freely available software WhisperX (57). In order to pseudonymize the data, the members of the study team listen to the therapy session and check the transcript at the same time. In this way it is not only possible to delete personal data but also keep track of transcription errors. Furthermore, this also enables the correction of errors in speaker diarization which will be relevant for data analysis. Transcripts are also edited according to predefined rules to ensure consistency, including standardized notation for stuttering and speech disfluencies, clear indication of overlapping speech, flagging of incomprehensible sections, and documentation of interruptions such as third parties entering. By doing this we are left with not only a pseudonymized audio track but also with a high quality pseudonymized and diarized transcript. Afterwards, the transcripts are ready for use with e.g., locally operating LLMs to assess their capability to distinguish between patient groups and evaluate symptom severity. The audio tracks are ready for feature extraction with the open-source toolkit openSMILE (56). Extracted audio features will serve as the basis for subsequent calculations, e.g., correlations with specific symptoms as well as general severity scores. Details of the unimodal and multimodal analyses are provided in the Statistical Methods section.

### Confidentiality

A dedicated data security concept for this study was developed in close collaboration with the local data protection officer, fully compliant with the EU General Data Protection Regulation [GDPR, Regulation (EU) 2016/679] and the German Federal Data Protection Act (Bundesdatenschutzgesetz, BDSG). The measures ensure both technical and organizational protection of personal data throughout the project. Key provisions include strict pseudonymization and, where feasible, anonymization of all personal data before processing. Access to sensitive data is restricted through a role-based authorization system, and all project staff are trained in privacy and IT security procedures. Data transfers are conducted via encrypted communication channels (TLS/SSL), and stored data is protected using state-of-the-art encryption algorithms. Routine system audits, monitoring, and regular penetration testing are implemented to detect and mitigate vulnerabilities. Redundant backups are encrypted and stored in physically secure environments, with access strictly controlled. Detailed data usage logs and audit trails are maintained to record all data access, modifications, and deletions. The network infrastructure is protected by firewalls and intrusion detection systems, and written informed consent is mandatory for all data subjects. These measures collectively ensure the confidentiality, integrity, and availability of research data, safeguarding data subjects' rights according to EU and German law. Specifically, we implemented the following measures to ensure data security. For every enrolled patient a participation ID will be generated via SecuTrial, a software for managing and conducting clinical trials, offering data collection, management, and analysis with high security and quality standards. One secured document which is saved locally in the University hospital Frankfurt contains the assigned patient identities to the SecuTrial-IDs. All confidential data will be stored exclusively in the university hospital Frankfurt. The questionnaire data will be transferred to SecuTrial and stored under the assigned participant ID. The raw audio files and their transcripts are then pseudonymized by a member of the study group. This involves deleting elements that can be clearly attributed to the patient or the clinician, such as names, addresses, institutions, or other personal information. All files are pseudonymized in compliance with data protection regulations and stored under the assigned participants ID. Password-protected Excel file (256-bit key) with the association of real names is stored in the hospital network. Only designated study personnel with identifiable names have knowledge of this password. Access to the system is logged in a log file.

## Statistical methods

### Statistical methods for primary and secondary outcomes

We will employ a zero-shot prompting approach with OpenAI's recently released GPT-OSS (Generative Pretrained Transformer—Open Source Software) series models, which are available under the permissive Apache 2.0 license and have demonstrated strong performance on reasoning tasks relevant to clinical assessment. We have selected these models for several methodological and practical reasons:

First, their open-weight nature ensures full transparency and reproducibility of our analyses. Second, their mixture-of-experts architecture enables efficient deployment on clinical infrastructure with limited computational resources. Third, their documented performance on complex reasoning tasks, including healthcare-related queries, makes them well-suited for psychiatric assessment applications.

Our primary analytical comparison will involve two model sizes from the GPT-OSS family: GPT-OSS-120B (116.8 billion total parameters with 5.1 billion active parameters per token) and GPT-OSS-20B (20.9 billion total parameters with 3.6 billion active parameters per token) (58). This comparison directly addresses a critical question for clinical deployment: whether larger, more capable models provide sufficient performance advantages to justify their greater computational demands, or whether smaller, more efficient models can achieve comparable diagnostic accuracy. The GPT-OSS-120B model has been shown to achieve near-parity with state-of-the-art reasoning models on standard benchmarks while running efficiently on a single 80GB GPU, whereas GPT-OSS-20B delivers comparable performance to advanced compact models while requiring only 16GB of memory, making it feasible for deployment on standard clinical workstations or even edge devices.

For each model size, we will systematically evaluate two distinct inference configurations defined by the temperature hyperparameter, which controls the randomness of model outputs. We will test a conservative configuration (temperature = 0.1) designed to prioritize consistency and clinical reliability by producing more deterministic responses, and a rather liberal configuration (temperature = 0.8). In the NLP literature the effects of temperature are well represented but systematic clinical applications have yet to be done to our knowledge. Accuracy in multiple choice question answering tasks show high consistency in temperature ranging between 0 and 1, but an expansion to other problem types is recommended (59). This strategic selection of hyperparameters reflects the clinical tension between reproducibility critical for diagnostic applications where consistency across assessments is paramount and the risk of highly inconsistent outcomes that could potentially lead to incorrect diagnoses and treatment recommendations. In addition, we apply top-p (nucleus) sampling with a cumulative probability threshold of 0.9 to each analysis. This setting restricts sampling to the smallest set of tokens whose cumulative probability mass is at least 0.9, balancing output diversity with coherence.

This yields a 2 × 2 factorial design with four experimental conditions: 1. GPT-OSS-120B with temperature 0.1, 2. GPT-OSS-120B with temperature 0.8, 3. GPT-OSS-20B with temperature 0.1, and 4. GPT-OSS-20B with temperature 0.8. We will evaluate each condition's performance on primary outcomes including general diagnostic classification accuracy and suicidality detection, severity score prediction (correlation with standardized clinical measures), and clinical utility metrics such as sensitivity, specificity, and positive/negative predictive values. All model versions, hyperparameters, and inference code will be fully documented and made available to ensure complete reproducibility.

Concurrent with text-based analysis using GPT-OSS models, we will extract acoustic features from therapy session recordings using openSMILE (version 3.0) (56) with the eGeMAPSv02 feature set (60), which includes validated markers known to associate with psychiatric conditions. The eGeMAPSv02 feature set comprises 88 acoustic features encompassing spectral (Mel-frequency cepstral coefficients), glottal (fundamental frequency, jitter, shimmer), and prosodic characteristics (energy, speech rate, voice quality measures such as harmonics-to-noise ratio). These features will be extracted at the frame-level (10-millisecond intervals) and subsequently aggregated using statistical functions (mean, standard deviation, minimum, maximum, percentiles) to produce session-level representations comparable to clinical assessment timeframes.

Our overarching analytical strategy employs a three-tier statistical framework designed to characterize how textual, acoustic, and combined modalities contribute to clinical prediction:

Tier 1: Unimodal Baseline Analysis. We will analyze each modality independently to establish baseline predictive performance before considering fusion. For the text modality, we will conduct two-way mixed-effects logistic regression with outcomes (diagnostic classification or severity degree) regressed on the fixed effects of model size and temperature, with random intercepts for participant and session. Effect sizes will be reported as odds ratios with 95% confidence intervals. For the acoustic modality, we will conduct mixed-effects linear regression using a reduced set of the most clinically relevant acoustic features selected *a priori* based on prior literature associations with depression and suicidality, including pitch variability, energy measures, MFCC and jitter as predictors of continuously scored clinical outcomes (e.g., severity scores from standardized rating scales) (60, 61).
Tier 2: Factorial Interaction Analysis for 2 × 2 Design. For text-based predictions, we will formally test the 2 × 2 factorial design using mixed-effects ANOVA to partition variance according to main effects (model size, temperature) and their interaction. Specifically, we will model primary outcome (e.g., predicted depression severity) as a function of fixed effects for model size (GPT-OSS-120B vs. 20B), temperature (0.1 vs. 0.8), and their interaction (Model Size × Temperature), with random intercepts for participants and sessions to account for the hierarchical data structure. This approach allows us to determine whether inference strategy recommendations (conservative vs. liberal temperature) can be applied consistently across model sizes, or whether optimization differs by model choice. If a significant Model Size × Temperature interaction emerges, it would suggest that clinical deployment requires model-specific tuning. Conversely, absence of interaction would support unified recommendations applicable regardless of computational constraints. We will report partial eta-squared (*η*²p) effect sizes with 95% confidence intervals for each factorial term.
Tier 3: Multimodal fusion and Late-Fusion Integration. To evaluate how textual and acoustic information combine for clinical prediction, we will employ a structured late-fusion approach. For each of the four 2 × 2 factorial conditions, we will generate separate predictions from the GPT-OSS models (text modality) and from a logistic regression model trained on acoustic features (acoustic modality). We will then integrate these predictions through weighted averaging, with weights determined via cross-validation (80% training, 20% held-out validation) on a stratified subset of sessions. This approach maintains interpretability—each modality's contribution can be quantified—and avoids the architectural complexity that would introduce uncontrolled hyperparameter flexibility incompatible with pre-registration.

We will quantify multimodal benefit by comparing fusion performance (AUC, sensitivity/specificity at clinically relevant decision thresholds) against the best unimodal baseline. If fusion provides incremental improvement beyond single modalities, we will use mixed-effects logistic regression with interaction terms to test whether the fusion benefit depends on model size or temperature setting. This tests the hypothesis that acoustic information provides complementary information that enhances text-based predictions uniformly, or whether certain model configurations better leverage multimodal information.

Statistical rigor and assumptions testing. For all mixed-effects models, we will verify assumptions including normality of residuals (Shapiro–Wilk test on standardized residuals), homogeneity of variance (Levene's test), and absence of multicollinearity (variance inflation factors < 5 for predictors). Violations of normality or homogeneity will be addressed through appropriate transformations (e.g., Box-Cox for acoustic features) or robust variance estimation. Missing data will be handled via maximum likelihood estimation under a missing-at-random assumption, standard for mixed-effects models.

Multiple comparisons within the 2 × 2 factorial framework will be controlled via Bonferroni correction applied within each outcome domain (text-based prediction, acoustic baseline, fusion). We will test all fixed effects (model size main effect, temperature main effect, Model Size × Temperature interaction) at *α* = 0.05 (two-tailed). *post-hoc* comparisons between specific factorial cells (e.g., testing whether GPT-OSS-120B at temperature 0.8 outperforms GPT-OSS-20B at temperature 0.1) will be pre-specified and corrected for multiple comparisons.

This simplified, focused approach ensures that our study adheres to pre-registration principles, prevents *post-hoc* methodological flexibility, and provides a reproducible framework that other research groups can directly replicate or build upon.

### Plans to give access to the full protocol, participant level-data and statistical code

Access to the full protocol, participant-level data, and statistical code will be restricted and carefully controlled. Researchers interested in accessing these materials must submit a formal application, which will be subject to review by the Institutional Review Board (IRB) for local data processing. This process is designed to ensure the protection of sensitive information and maintain the integrity of the research. It is important to note that, in accordance with the decision of the data security board at the University of Frankfurt, no data can be shared outside of this controlled environment consisting of the study team in Frankfurt and selected members of the study team in Darmstadt.

## Oversight and monitoring

### Composition of the coordinating center and trial steering committee

Director of Studies are PD Dr med. Oliver Grimm (OG) & Prof. Dr. Andreas Reif (AR) from the Department of Psychiatry, Psychosomatics and Psychotherapy, University Hospital Frankfurt. Further, the UKP Lab headed by Prof. Dr. Iryna Gurevych (IG) will cooperate and is responsible for the processing of the data, feature extraction and building of an NLP/multimodal model. The trial steering committee consists of OG, AR, and IG, along with a senior statistician and an independent expert in psychiatry research. This committee will oversee the overall conduct of the study, monitor its progress, and make key decisions regarding the trial's direction and any necessary protocol amendments.

### Composition of the data monitoring committee, its role and reporting structure

According to German law, no formal data monitoring committee is needed for a study without therapeutic intervention.

### Plans for communicating important protocol amendments to relevant parties (e.g., trial participants, ethical committees)

Protocol modifications will firstly be sent to the central and local ethics committees. Once changes are approved, currently enrolled patients will be asked to sign the newest version of the informed consent form accordingly.

## Dissemination plans

Study results will be communicated to the scientific community and general public via publications.

## Discussion

The SPEAK-SAFE study protocol presents a novel approach to utilizing Natural Language Processing (NLP)/multimodal methods for analyzing therapist-patient conversations in psychiatric care. This discussion addresses key aspects of the study design, potential challenges, and implications for future research and clinical practice.

The sensitive nature of the conversational data collected in this study necessitates robust privacy measures. Our study represents an initial feasible approach to this matter. Future research should further address these issues to optimize data collection while ensuring adequate implementation of data security. Collaboration with institutions like ATHENE will be instrumental in developing innovative and secure solutions for mental health research.

The integration of cutting-edge cybersecurity measures not only enhances the robustness of our data protection protocols but also sets a precedent for future studies in this field. This interdisciplinary approach, combining mental health research with advanced cybersecurity practices, paves the way for more secure and efficient data handling in sensitive research areas.

In addition to the challenges arising from the strict data protection guidelines, recording therapy sessions presents several technical challenges that could impact data quality. There are indications that the quality of the final results is more negatively affected by poor audio quality rather than computational issues (62). Background noise, variations in speech volume, and acoustic properties of the recording environment are crucial influencing factors which may affect transcription accuracy and overall data quality in non-laboratory settings (63). To mitigate these issues, we have implemented quality assurance strategies, including the use of high-quality recording equipment and careful placement of microphones. Nevertheless, some degree of data loss or inaccuracy may be unavoidable, potentially impacting the AI model's performance.

What accuracy should be expected from an AI-based suicide evaluation? Large language models demonstrate strong performance in detecting depression and suicide risk from clinical narratives, with recent real-world validation substantially informing expectations for psychotherapy transcripts. In a recent cross-sectional study of 100 admission notes from a psychiatric hospital, a German-fine-tuned Llama-2 model achieved 87.5% accuracy, 83.0% sensitivity, and 91.8% specificity in identifying suicidality status against expert ground truth, demonstrating robust performance across varied prompting strategies while preserving data privacy (64). To contextualize these capabilities, human interrater reliability in assessing mental health from dialogue provides an essential benchmark. Unstructured suicide risk assessment shows substantially lower concordance: psychotherapists demonstrate Gwet's AC₁ = 0.44 with only 58% identical risk classifications, while psychology students achieve AC₁ = 0.35 (65). Unstructured psychiatric diagnosis using clinical interviews yields *κ* = 0.24–0.43 (fair at best), compared to *κ* = 0.75 (substantial) when structured protocols guide assessment (66). This empirical gap is instructive: current clinical practice relies on unstructured judgment with relatively poor reliability, particularly for suicide risk estimation. Consequently, LLM-assisted assessment of suicidality in psychotherapy transcripts is already useful potentially when achieving only modest accuracy performance.

The dialogic nature of therapy sessions introduces complexities in transcription and analysis. Colloquialisms, pauses, overlapping speech, and non-verbal cues are integral to therapeutic communication but challenging to capture and analyze (67). Our multimodal approach aims to address these challenges by incorporating advanced speech recognition and sentiment analysis techniques. However, the nuances of therapeutic dialogue may still pose difficulties in accurate interpretation, necessitating ongoing refinement of our multimodal models.

The SPEAK-SAFE study utilizes both structured (questionnaires) and unstructured (conversation recordings) data. While structured data offers standardized, easily quantifiable information, unstructured data provides rich, contextual insights into patient-therapist interactions (68). Our decision to focus on conversational data stems from its potential to capture subtle linguistic markers of mental health states that may not be evident in structured assessments. Our aim is to predict occurring changes in the structured data, what we consider as ground truth, by using the rich, unstructured speech data.

A limitation of the current study design is the lack of long-term follow-up to assess the predictive accuracy of the NLP/multimodal model over time. This has been done so far by only few studies (36). While our approach provides valuable insights into immediate risk assessment, the dynamic nature of mental health conditions necessitates longitudinal evaluation. Future research should incorporate extended follow-up periods to validate the model's long-term predictive capabilities and assess its utility in tracking treatment progress.

The use of AI in mental healthcare raises important ethical considerations. Potential biases in NLP/multimodal models, particularly regarding cultural and linguistic diversity, must be carefully addressed (69). Moreover, the integration of AI-based tools in clinical decision-making necessitates a balance between technological assistance and human clinical judgment. Our study emphasizes the importance of human oversight in interpreting AI-generated insights, recognizing that these tools should augment rather than replace clinical expertise.

The SPEAK-SAFE study has significant potential to enhance mental health care. By providing clinicians with AI-assisted risk assessment tools, we aim to improve the accuracy and efficiency of suicide risk evaluation (34). This could lead to more timely interventions and personalized treatment strategies. However, the successful integration of such tools into clinical workflows requires careful consideration of user acceptance, training needs, and potential impacts on the therapeutic relationship.

Building on the SPEAK-SAFE study, future research should explore the application of our AI models to a broader range of mental health conditions. Additionally, investigating the integration of multimodal data, including physiological measures and social media activity, could enhance the model's predictive capabilities (13). Longitudinal studies examining the long-term impact of AI-assisted care on patient outcomes are also crucial for validating the clinical utility of this approach. We plan to augment our data collection with smartphone-based ecological momentary assessment (EMA) to allow outcome prediction.

In conclusion, the SPEAK-SAFE study represents a significant step towards leveraging AI in mental health care. While challenges remain in data collection, analysis, and ethical implementation, the potential benefits in improving risk assessment and patient care are substantial. Continued research and interdisciplinary collaboration will be essential in refining these approaches and translating them into clinical practice.

## Trial status

Current Protocol version 6, 08.04.2025. Recruitment began on 1st of November 2023. Data collection will continue until further notice without end date.
